# Optimizing recovery of marginal donor hearts with low ejection fraction: A national retrospective study from Switzerland

**DOI:** 10.1016/j.jhlto.2025.100411

**Published:** 2025-10-17

**Authors:** Raphaël Giraud, Selina Adam, Benjamin Assouline, Franziska Beyeler, Roger Hullin, Karim Bendjelid, Franz Immer

**Affiliations:** a Intensive Care Division, Geneva University Hospitals, Geneva, Switzerland; b Faculty of Medicine, University of Geneva, Geneva, Switzerland; c Geneva Hemodynamic Research Group, Faculty of Medicine, University of Geneva, Geneva, Switzerland; d Latin Organ Donation Program, University Hospitals of Geneva, Geneva, Switzerland; e Faculty of Medicine, University of Bern, Bern, Switzerland; f Swisstransplant, the Swiss National Foundation for Organ Donation and Transplantation, Bern, Switzerland; g Cardiology, Cardiovascular Department, University Hospital and University of Lausanne, Lausanne, Switzerland

**Keywords:** heart transplantation, marginal donor hearts, left ventricular ejection fraction, echocardiography, coronary angiography, donor management

## Abstract

**Background:**

In Switzerland, heart transplantation is limited by donor availability. Marginal donor hearts—characterized by older age, comorbidities, or reduced left ventricular ejection fraction (LVEF)—are often declined despite being structurally normal. Since impaired LVEF may be reversible, optimized donor management could improve cardiac function and transplant eligibility.

**Methods:**

This retrospective cohort study analyzed 415 brain-dead donors (2017–2021) from the Swiss Organ Allocation System. Donors were categorized as optimal (LVEF ≥50%) or marginal (LVEF 15–49%), irrespective of structural abnormalities, to focus on a modifiable functional parameter. Predictors of heart transplantation were assessed using univariate and multivariable logistic regression. Additional analyses evaluated hemodynamic monitoring, diagnostic strategies, management duration, and functional recovery based on serial echocardiography.

**Results:**

Among 415 donors, 287 were optimal and 62 marginal. Transplantation rates were higher in optimal hearts (62.7% vs. 23.6%, p < 0.001). LVEF ≥50% independently predicted transplantation (adjusted OR = 4.56; 95% CI: 2.34–8.89; p < 0.001), together with younger age, lower norepinephrine dose, central monitoring, and repeated echocardiography. Marginal hearts demonstrated significant improvement in LVEF after optimized management (p < 0.0001), suggesting that reversible dysfunction can be mitigated through tailored hemodynamic and pharmacologic strategies.

**Conclusions:**

Optimized donor management can restore left ventricular function in marginal hearts and enhance transplant eligibility. Standardized national guidelines may help expand the donor pool and improve transplantation outcomes.

## Graphical abstract

**Figure:**
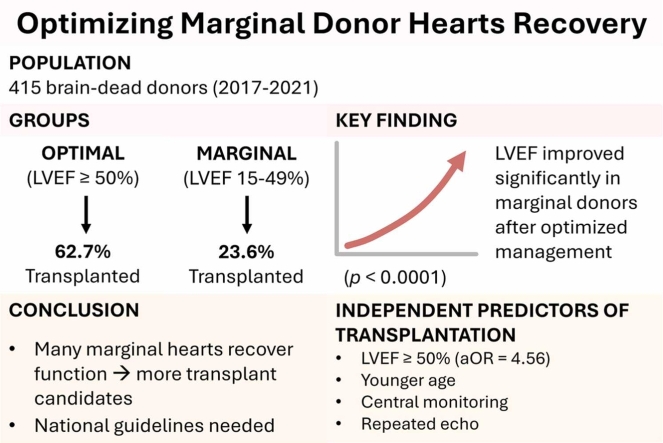

## Background

Heart transplantation is recognized as the gold-standard treatment for patients with end-stage heart failure, substantially improving both survival rates and quality of life.^1^ Despite its success, the procedure is highly restricted by the lack of suitable donor hearts, a problem that continues to be increasingly prevalent around the world, and especially significant in Switzerland.^2^ In 2021, the large gap between the number of patients awaiting heart transplantation (126) and the number of heart transplantations (33) performed in Switzerland calls for solutions to increase donor rates.

Strict criteria often lead to the rejection of hearts with decreased left ventricular ejection fraction (LVEF), even when these hearts are structurally normal.^3^ The clinical conditions resulting from brain death (BD), such as severe hormonal and hemodynamic decompensation, catecholaminergic stress, and systemic inflammation, often cause myocardial dysfunction, resulting in additional decrements in LVEF.^4^ Nevertheless, these dysfunctions could be reversed in many cases, with proper management interventions.^5^ This reflects the underutilization of marginal donor hearts, which have sufficient potential of recovery as well as utilization for transplantation.^6^

To better address the clinical question of whether temporary systolic dysfunction in brain-dead donors (DBD) is a barrier or an opportunity for transplantation, we focused specifically on LVEF as a potentially reversible criterion. In contrast to structural cardiac pathologies, such as valvular disease or coronary artery disease (CAD), LVEF can respond to hemodynamic optimization and hormonal resuscitation, making it a clinically actionable parameter during donor management.

Donor management protocols improvements have brought new dynamic evaluation criteria and therapeutic measures, potentially being a promising resource to work on marginal hearts utilization.^7^ Improved donor management support might even enable the donor pool to be extended with no detrimental impact on transplantation results.^8^ The present study aims to identify the barriers that impede the use of marginal hearts in Switzerland, the specific management differences between optimal and marginal donors and to present the evidence-based interpretation along with operational recommendations to deal with these issues.

Based on detailed analysis of the data from the Swiss Organ Allocation System, this study aims to seeks to bridge the gap between clinical restrictions and increasing demand for donor hearts. It is the first to evaluate functional recovery in marginal donor hearts using serial echocardiography and structured optimization within a nationally coordinated allocation system that allows for prolonged management time. The study also considers the systemic implications of using marginal hearts for transplantation, particularly in the setting of worldwide organ shortage and in pursuit of a more equitable organ allocation policy.

In this study, we deliberately chose to classify donor hearts based solely on left ventricular systolic function (LVEF), as this is the only donor cardiac parameter that can potentially improve during intensive care optimization. Other structural features, such as CAD, valvular disease, or hypertrophy, are not amenable to short-term reversal and were therefore not included in our definition of marginality.

## Methods

### Study setting and data collection

This retrospective observational study analyzed all DBD hearts recorded in the Swiss Organ Allocation System between 2017 and 2021. The cohort included 415 donors aged 18 to 70 years, whose hearts were classified as marginal (LVEF 15%-49%) or optimal (LVEF ≥50%). Other structural abnormalities, such as CAD, valvular disease, or left ventricular hypertrophy, were not used to define marginality, as these were not considered potentially reversible during donor optimization. Donors with LVEF <15%, deemed nontransplantable by clinical standards, were excluded. Hearts without pretransplant echocardiography were considered ineligible for allocation and analyzed separately.

Collected variables included age, sex, cause of BD, comorbidities, echocardiographic data, and donor management parameters: vasopressor use, diagnostic timing, and optimization duration. Optimization, defined as the period from BD to procurement, involved targeted interventions to improve organ function. As per Swiss protocols, this phase may last up to 72 hours and includes hemodynamic stabilization (e.g., mean arterial pressure >60 mm Hg, cardiac index >2.4 liter/min/m²), central monitoring (central venous pressure [CVP] or pulmonary artery catheter), hormonal therapy (triiodothyronine, corticosteroids, vasopressin), insulin for glycemic control (target 120-180 mg/dl), and repeated diagnostics (echocardiography, coronary angiography if indicated).^9^ No donors in this study were managed using ex vivo perfusion systems such as the Organ Care System (OCS).

Structurally normal hearts were defined as lacking significant valvular pathology, myocardial hypertrophy, right ventricular dysfunction, or known cardiomyopathy, based on echocardiography and angiography (if available).

Anoxic injury was diagnosed based on clinical history of prolonged hypoxia or cardiac arrest, radiological evidence of diffuse cerebral edema, and/or electroencephalography inactivity.

These procedures were designed to improve the functional recovery, with special emphasis on marginal hearts.

The study was in compliance with ethical standards for research using organ donation material in Switzerland, and special care was taken to ensure data confidentiality. In the opinion of the Ethics Committee of the Canton of Bern (Req-2025-00514), the planned project is not subject to the Swiss Human Research Act and thus does not need a prospectively obtained approval from the responsible ethics committee.

The purpose of the study was to identify clinical and logistical parameters associated with the utilization of donor hearts for transplantation, focusing on an efficient use of marginal donor hearts under logistical and ethical restrictions in the Swiss allocation system.

### Statistical analysis

The primary end-point was the relationship between the LVEF and heart transplant. Donor hearts were characterized as marginal (LVEF 15%-49%) or optimal (LVEF 50%). Comparison of categorical variables such as LVEF group and the status of transplantation was done using Pearson’s chi-square test. Non-Gaussian distributed continuous variables (time to first echocardiogram, peak norepinephrine [NE] dose) were compared with the Mann-Whitney U test.

Continuous variables are expressed as medians with interquartile ranges (IQR) and categorical variables as frequencies (%). In donors undergoing serial echocardiography, changes in LVEF were assessed using repeated-measures 2-way analysis of variance with time (first vs last echocardiogram) and functional trajectory (improvement vs decline) as fixed factors. Post hoc pairwise comparisons were performed to investigate between-group differences.

Independent predictors of transplantation were determined with a multivariable logistic regression model. The included variables were sex, age, LVEF category, cause of death, dose of NE, central hemodynamic monitoring, repeated echocardiograms, coronary angiography, and time to optimization. Results are presented as odds ratios with 95% confidence intervals (CIs) and *p-*values.

Sensitivity analyses investigated the relationship between serial echocardiography and LVEF recovery. Subgroup analyses were performed in the following donor groups. Data were analyzed by IBM SPSS Statistics Version 28.0, and the level of significance was set at *p* < 0.05.

## Results

### Donor demographics

**Figure 1:**
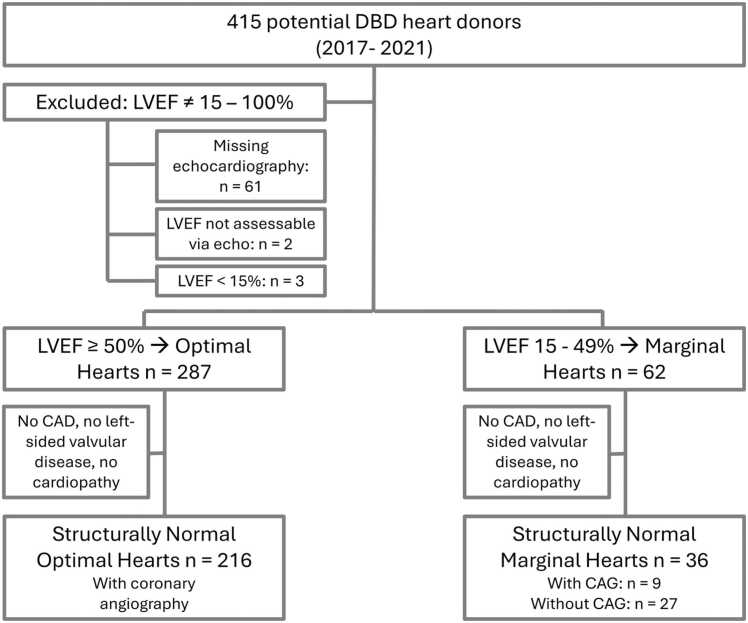
Flowchart of study design. From 577 DBD donors between January 1, 2017 and December 31, 2021, a total of 415 met the study inclusion criteria. Donors with no echocardiography (*n* = 61), nonassessable LVEF (*n* = 2), or LVEF <15% (*n* = 3) were excluded from the marginal vs optimal heart analysis. Structurally normal hearts were defined only within the optimal and marginal groups and were further evaluated with or without coronary angiography (CAG). Structurally normal hearts were defined as those without significant valvular disease, myocardial hypertrophy, right ventricular dysfunction, or known cardiomyopathy. CAD, coronary artery disease; DBD, brain-dead donors; LVEF, left ventricular ejection fraction.

Between 2017 and 2021, 415 DBD hearts were evaluated. Sixty-six were excluded: 61 lacked echocardiography, 2 had unassessable LVEF, and 3 had LVEF <15%. The remaining 349 hearts were analyzed: 287 (82.2%) classified as optimal (LVEF ≥50%) and 62 (17.8%) as marginal (LVEF 15%-49%) (Table 1, Figure 1).

**Table 1:** Overview of the Donor Collective (*n* = 415)

Parameter	Total	LVEF ≥50%	LVEF 15%-49%	LVEF <15%	No echo	No LVEF in echo
Patients, *n* (%)	415 (100.0)	287 (69.2)	62 (14.9)	3 (0.7)	61 (14.7)	2 (0.5)
Tx, *n* (%)	194 (46.7)	180 (62.7)	14 (23.6)	0 (0.0)	0 (0.0)	0 (0.0)
No Tx, *n* (%)	221 (53.3)	107 (37.3)	48 (77.4)	3 (100.0)	61 (100.0)	2 (100.0)
Rejected, *n* (%)	120 (54.3)	85 (79.4)	32 (66.6)	0 (0.0)	2 (3.3)	1 (50.0)
Not offered, *n* (%)	101 (45.7)	22 (20.6)	16 (33.4)	3 (100.0)	59 (96.7)	1 (50.0)

Abbreviations: LVEF, left ventricular ejection fraction; Tx, transplantation.

Median donor age was 52 years (IQR 36-60), with no significant difference between groups. However, gender distribution differed (*p* = 0.0457), with more male donors in the optimal group (56.4%) and more female donors in the marginal group (58.1%) (Table 2). Marginal donors had significantly lower weight (67 vs 76 kg, *p* = 0.0022) and body mass index (23.9 vs 24.7 kg/m², *p* = 0.009), while height differences were not significant.

**Table 2:** Baseline Data of the Central Subgroups (Marginal and Optimal Hearts) of the Total Cohort

Variable	Total (*n* = 415)	Optimal (LVEF ≥50%) (*n* = 287)	Marginal (LVEF 15%-49%) (*n* = 62)	*p*-value
Gender, *n* (%)				0.0457
Male	236 (56.9)	162 (56.4)	26 (41.9)	
Female	179 (43.1)	125 (43.6)	36 (58.1)	
Age [years], med (IQR)	52 (36-60)	51 (36-59)	52 (29-60)	0.6073
Weight [kg], med (IQR)	75 (65-85)	76 (66-87)	67 (60-75)	0.0022
Height [cm], med (IQR)	172 (165-180)	173 (165-180)	170 (164-175)	0.1125
BMI [kg/m^2^], med (IQR)	24.8 (22.6-27.8)	24.7 (22.8-27.8)	23.9 (21.9-26)	0.009
Hypertension, *n* (%)				0.5027
No	304 (75.6)	217 (77)	50 (82)	
Yes	98 (24.4)	65 (23)	11 (18)	
Diabetes, *n* (%)				0.8891
No	371 (90.9)	262 (91.9)	58 (95.1)	
Yes	37 (9.1)	23 (8.1)	3 (4.9)	
Cause of death, *n* (%)				0.0034
Anoxia	142 (34.2)	79 (27.5)	29 (46.8)	
Cerebral disease	12 (2.9)	7 (2.4)	2 (3.2)	
Cerebral hemorrhage	162 (39)	117 (40.8)	25 (40.3)	
Cerebral trauma	90 (21.7)	79 (27.5)	5 (8.1)	
Stroke	3 (0.7)	2 (0.7)	0 (0.0)	
Other causes	6 (1.4)	3 (1)	1 (1.6)	

Abbreviations: BMI, body mass index; IQR, interquartile ranges; LVEF, left ventricular ejection fraction.

The 2 LVEF-based donor groups did not differ significantly with regard to structural cardiac abnormalities. Specifically, no statistically significant differences were observed between the groups in the prevalence of arterial hypertension (*p* = 0.066), valvular cardiopathy (*p* = 0.281), or myocardial hypertrophy (*p* = 0.075). CAD showed only a borderline significance (*p* = 0.047). These results support our focus on LVEF as the key functional variable distinguishing the groups.

Comorbidity rates were similar, though hypertension and diabetes were slightly less prevalent among marginal donors. Causes of BD varied significantly (*p* = 0.0034): anoxia was more common in marginal donors (46.8% vs 27.5%), while cerebral trauma was more frequent in optimal donors (27.5% vs 8.1%).

Among structurally normal hearts, 216 were optimal and 36 marginal; only 9 marginal hearts underwent coronary angiography. Transplantation rates differed markedly: 62.7% for optimal vs 23.6% for marginal hearts (Table 1), reflecting both functional and clinical disparities.

### Transplantation rates and disqualification reasons

Transplantation was significantly more frequent among optimal hearts (62.7%, 180/287) than marginal ones (23.6%, 14/62; *p* < 0.001). Among the 107 optimal hearts not transplanted, the most common disqualification reason was CAD (29.9%), followed by other medical conditions (23.4%), cardiac hypertrophy (12.2%), right ventricular failure or cardiomyopathies (8.4%), and lack of a suitable recipient (7.5%). Additional causes included valvular disease (5.6%), anoxic damage (6.5%), and advanced donor age (4.7%). Only 1 optimal heart (0.9%) was excluded for poor LVEF on final assessment.

In marginal hearts, persistent LVEF dysfunction accounted for 39.6% (19/48) of nontransplant cases. Other reasons included CAD (14.5%), hypertrophy (10.4%), severe anoxic injury (10.4%), and cardiomyopathies such as dilated forms or sarcoidosis (10.4%). A further 14.7% were disqualified due to unspecified medical issues.

These data underscore the complexity of marginal donor evaluation (Table 3). While LVEF recovery may occur, irreversible structural or systemic pathology often remains a barrier to transplantation. Enhanced assessment protocols—including advanced imaging and repeated functional evaluation—may improve the identification of reversible impairments and support more effective selection of transplantable marginal hearts.

**Table 3:** Overview of Reasons for Rejection in Heart Transplantation for Optimal and Marginal Hearts

Reason for rejection	Optimal hearts (*n* = 107)	Marginal hearts (*n* = 48)	*p*-value
Valvular cardiopathy, *n* (%)	6 (5.6)	3 (6.3)	1.0000
Unknown, *n* (%)	1 (0.9)	1 (2.1)	0.5248
Poor LVEF, *n* (%)	1 (0.9)	19 (39.6)	<0.0001
Coronary heart disease, *n* (%)	32 (29.9)	7 (14.5)	0.0468
No compatible recipient, *n* (%)	8 (7.5)	1 (2.1)	0.2757
Hypertrophy, *n* (%)	13 (12.2)	5 (10.4)	1.0000
Anoxia, *n* (%)	7 (6.5)	4 (8.3)	0.7392
Other medical reasons, *n* (%)	25 (23.4)	3 (6.3)	0.0120
Other cardiological diseases, *n* (%)	9 (8.4)	5 (10.4)	0.7638
Age, *n* (%)	5 (4.7)	0 (0.0)	0.3249

Abbreviation: LVEF, left ventricular ejection fraction.

### Echocardiographic findings and recovery

**Figure 2:**
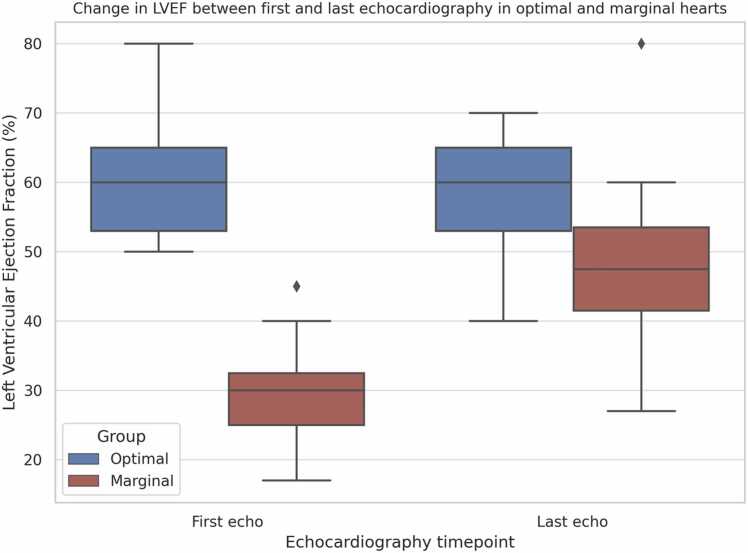
Graphical representation of the change in LVEF between the first and last echocardiography in optimal and marginal donor hearts. LVEF, left ventricular ejection fraction.

Echocardiography proved essential for assessing functional recovery. Among marginal hearts, 15 cases (24.2%) showed significant LVEF improvement during optimization, increasing from 29.9% to 48.2% (*p* < 0.0001) (Table 4). Optimal hearts remained stable, with no significant changes (Figure 2).

**Table 4:** Comparative Analysis of Donor Hearts With Serial Echocardiographic Evaluations, Focusing on Changes in Left Ventricular Ejection Fraction

Variables	LVEF improved (optimal) (*n* = 5)	LVEF declined (optimal) (*n* = 10)	LVEF improved (marginal) (*n* = 15)	Statistical comparison (improved optimal vs marginal) *p*-value
LVEF first echo [%], med (IQR)	50 (50-57)	65 (60-66)	30 (25-37)	<0.0001
LVEF last echo [%], med (IQR)	64 (55-65)	60 (45-65)	48 (42-55)	<0.0001
CAD, yes *n* (%)	2 (40.0)	6 (60.0)	4 (26.7)	0.6126
ROSC [min], med (IQR)	50 (50-50)	15 (10-15)	23 (13-40)	<0.0001
NA peak [mcg/kg/min], med (IQR)	0.235 (0.19-0.375)	0.145 (0.117-0.229)	0.167 (0.1-0.475)	<0.0001
NA last [mcg/kg/min], med (IQR)	0.141 (0.09-0.375)	0.101 (0.05-0.154)	0.119 (0.05-0.3)	<0.0001
Central monitoring, no *n* (%)	3 (60.0)	3 (30.0)	6 (40.0)	0.6169
Central monitoring, yes *n* (%)	2 (40.0)	7 (70.0)	9 (60.0)	0.6169
First CVP [mm Hg], med (IQR)	8 (5-10)	8 (5-11)	8 (8-9)	0.0841
Last CVP [mm Hg], med (IQR)	6 (5-6)	10 (5-10)	9 (8-11)	<0.0001
Transplanted, *n* (%)	3 (60.0)	6 (60.0)	6 (40.0)	0.6169
Rejected, *n* (%)	2 (40.0)	4 (40.0)	9 (60.0)	0.6169
Not offered, *n* (%)	0 (0.0)	0 (0.0)	0 (0.0)	

Abbreviations: CAD, coronary artery disease; CVP, central venous pressure; IQR, interquartile ranges; LVEF, left ventricular ejection fraction; NA, noradrenaline; NA last, last administered NA dose; NA peak, highest administered NA dose; ROSC, return of spontaneous circulation.

Among transplanted hearts, early post-transplant outcomes were available for 180 optimal and 14 marginal hearts. In marginal hearts, no episodes of primary graft dysfunction were reported at 30 days, and LVEF was >50% in 13 of 14 recipients. No significant difference in 30-day survival was noted between marginal and optimal donor hearts (*p* = 0.48).

Repeated-measures 2-way analysis of variance confirmed a significant interaction for LVEF over time in the marginal group (F = 21.11, df = 1.29, *p* < 0.0001), but not in optimal donors. Repeated echocardiography was more frequently used in marginal hearts (25.8%), particularly in those showing functional improvement, suggesting targeted reassessment in clinically evolving donors.

The median time from BD to heart procurement was 25.8 hours for marginal donors and 22.7 hours for optimal donors. No noncardiac organs were declined due to prolonged optimization in this cohort. Although specific metrics such as lactate levels or diuresis were not consistently documented, no negative impact on multiorgan procurement was observed.

Univariate analysis showed a significant association between repeated echocardiography and utilization for transplantation (*p* = 0.026), and this remained significant in multivariable regression (adjusted odds ratio = 2.12, 95% CI: 1.09-4.11, *p* = 0.026).

These findings highlight the importance of structured donor management and continuous functional reassessment in improving transplant eligibility, particularly among marginal donors.

### Management practices

Marginal hearts required higher catecholamine support, with median NE doses of 0.2 mcg/kg/min (IQR 0.1-0.375), exceeding the recommended threshold of 0.08 mcg/kg/min. This hemodynamic instability correlated with earlier echocardiographic assessment—31 hours post BD vs 36 hours for optimal hearts (*p* = 0.015).

In structurally normal hearts (Table 5), marginal donors showed greater NE use and a trend toward more frequent central hemodynamic monitoring. Although differences in CVP and monitoring did not reach significance, marginal hearts consistently required more intensive support ( Tables 5 and 6).

**Table 5:** Comparison of Structurally Normal Hearts

Parameter	Optimal (*n* = 216)	Marginal (*n* = 36)	*p*-value
>1 Serial echocardiography, *n* (%)	12 (5.6)	14 (38.8)	<0.001
CAD, *n* (%)			
No	100 (46.3)	27 (75.0)	0.002
Yes	116 (53.7)	9 (25.0)	
Resuscitation, *n* (%)			
No	134 (62.0)	16 (44.4)	0.066
Yes	82 (38.0)	20 (55.6)	
ROSC [min], med (IQR)	20 (13-33)	18 (10-40)	0.814
NA therapy, *n* (%)			
No	64 (29.6)	7 (19.4)	0.236
Yes	152 (70.4)	29 (80.6)	
NA >0.08 mcg/kg/min, *n* (%)			
No	49 (32.5)	5 (17.2)	0.124
Yes	102 (67.5)	24 (82.8)	
NA peak [mcg/kg/min], med (IQR)	0.126 (0.069-0.256)	0.2 (0.1-0.375)	0.059
NA last [mcg/kg/min], med (IQR)	0.077 (0.03-0.167)	0.082 (0.33-0.215)	0.511
Central monitoring, *n* (%)			
No monitoring	103 (47.7)	22 (61.1)	
Monitoring total	113 (52.3)	14 (38.9)	0.152
Including CVP	113 (52.3)	14 (38.9)	0.152
Including Swan-Ganz catheter	1 (0.5)	2 (5.6)	0.054
First CVP [mm Hg], med (IQR)	7 (6-10)	8 (8-11)	0.282
Last CVP [mm Hg], med (IQR)	7 (5-10)	9 (5-12)	0.252

Abbreviations: CAD, coronary artery disease; CVP, central venous pressure; IQR, interquartile ranges; LVEF, left ventricular ejection fraction; NA, noradrenaline; NA last, last administered NA dose; NA peak, highest administered NA dose; ROSC, return of spontaneous circulation.

Differences between optimal and marginal hearts are indicated with significance levels.

**Table 6:** Comparison of Structurally Normal Hearts, Divided Into Those With and Without Additional Coronary Angiography

Parameter	Structurally normal hearts with CAD (optimal)	Structurally normal hearts with CAD (marginal)	Structurally normal hearts without CAD (optimal)	Structurally normal hearts without CAD (marginal)	*p*-value
CAD, *n* (%)	116 (100.0)	9 (100.0)	0 (0.0)	0 (0.0)	N/A
Resuscitation, *n* (%)					
No	69 (59.5)	4 (44.4)	65 (65.0)	12 (44.4)	0.488/0.075
Yes	47 (40.5)	5 (55.6)	35 (35.0)	15 (55.6)	
ROSC [min], med (IQR)	15 (10-30)	10 (10-60)	23 (15-38)	20 (12-40)	0.914/0.763
NA therapy, *n* (%)					
No	28 (32.8)	4 (44.4)	26 (26.0)	3 (11.1)	0.717/0.125
Yes	78 (67.2)	5 (55.6)	74 (74.0)	24 (88.9)	
NA >0.08 mcg/kg/min, *n* (%)					
No	23 (29.5)	1 (20)	26 (35.6)	4 (16.7)	1.000/0.125
Yes	55 (70.5)	4 (80)	47 (64.4)	20 (83.3)	
NA peak [mcg/kg/min], med (IQR)	0.141 (0.067-0.257)	0.167 (0.14-0.167)	0.12 (0.07-0.24)	0.21 (0.1-0.448)	0.846/0.061
NA last [mcg/kg/min], med (IQR)	0.069 (0.035-0.178)	0.05 (0.027-0.088)	0.077 (0.03-0.141)	0.101 (0.042-0.264)	0.210/0.204
Central monitoring, *n* (%)					
No monitoring	53 (45.7)	7 (77.8)	50 (50.0)	15 (55.6)	N/A
Monitoring total	63 (54.3)	2 (22.2)	50 (50.0)	12 (44.4)	0.086/0.688
Including CVP	63 (54.3)	2 (22.2)	50 (50.0)	12 (44.4)	
Including Swan-Ganz Catheter	1 (0.9)	0 (0.0)	0 (0.0)	2 (7.4)	1.000/0.044
First CVP [mm Hg], med (IQR)	8 (6-9)	13 (9-17)	7 (5-10)	8 (7-10)	0.124/0.492
Last CVP [mm Hg], med (IQR)	7 (5-10)	13 (9-16)	7 (5-10)	8 (5-12)	0.149/0.498

Abbreviations: CAD, coronary artery disease; CVP, central venous pressure; IQR, interquartile ranges; LVEF, left ventricular ejection fraction; NA, noradrenaline; NA last, last administered NA dose; NA peak, highest administered NA dose; ROSC, return of spontaneous circulation.

Differences between optimal and marginal hearts are indicated with significance levels.

Levosimendan, a cardioprotective inotrope, was administered in only 1 case, indicating limited adoption despite potential benefits. This raises questions about its underutilization in marginal donor optimization.

Median management duration was 61 hours for marginal hearts vs 57 for optimal, suggesting suboptimal use of the full 72-hour window allowed in Swiss donor protocols.

## Discussion

This retrospective analysis of Swiss DBD donors demonstrates that hearts with reduced LVEF can recover during donor management and, in selected cases, be successfully utilized for transplantation. These findings support the concept that intensive care interventions may positively influence transplant eligibility, even in donors initially classified as marginal.

Our methodological choice to define marginality based on LVEF alone was deliberate and grounded in clinical relevance. Unlike fixed structural conditions, systolic dysfunction may respond to donor optimization protocols. In this context, we aimed to isolate LVEF as a potentially modifiable barrier to organ utilization, rather than confounding the analysis with unchangeable anatomical contraindications. This approach allows us to explore whether functional recovery during donor management can unlock the potential of hearts initially deemed unsuitable. Although recent guidelines (Copeland, 2023 #85) suggest a multidimensional assessment of donor heart quality, our classification was intentionally limited to LVEF. This approach reflects the pragmatic perspective of intensive care management, where improving systolic function through hemodynamic and pharmacologic interventions remains feasible, unlike correcting structural heart disease in the limited timeframe of donor care.

### The role of LVEF in optimized donor management and outcomes

Optimized donor management can improve marginal heart function and transplantation outcomes.^10^, ^11^, ^12^, ^13^, ^14^ This study found a strong association between LVEF improvement and transplant eligibility in Swiss donors (2017-2021, *p* < 0.001). Functional recovery occurred only in marginal hearts (*p* < 0.0001), reflecting the impact of targeted management strategies already in place. However, only 23% of marginal hearts were transplanted vs 62% of optimal hearts, suggesting restrictive LVEF thresholds remain a barrier.

Among transplanted hearts, only 38.9% of marginal hearts with recovered LVEF had structurally normal hearts, compared to 75.9% of optimal hearts. A large comparative study (Eurotransplant *n* = 8,714; United States *n* = 60,882) identified older age, comorbidities, reduced LVEF, and structural abnormalities as rejection predictors. Despite higher-risk donors, Eurotransplant had a higher acceptance rate than the United States (70% vs 44%).^15^

Bifulco et al reported higher early mortality in marginal heart recipients, though mid-term outcomes were similar.^16^ In contrast, this study observed better early function in structurally normal marginal hearts, with no early graft failure. These findings support extended optimization and reassessment to improve marginal heart utilization.

### Management of marginal and optimal donor hearts

Hess et al demonstrated that marginal donor hearts managed in specialized centers can yield outcomes comparable to optimal hearts from nonspecialized centers.^17^ This supports the value of targeted pretransplant optimization, especially for marginal hearts with coronary or valvular disease. In Switzerland, routine coronary assessment via angiography or cardiac CT sets national practice apart. Nonetheless, stress echocardiography may offer additional insight into contractile reserve in borderline cases.^18^

Bifulco et al identified renal dysfunction, ischemia time, and inotrope use as key risks for marginal grafts, reinforcing the need for structured management.^16^ Variability across Swiss centers highlights the need for standardized protocols.

While the Swiss Donation Pathway provides national guidance, practices differ. For example, ex vivo systems such as the OCS are used more often in Switzerland to offset prolonged ischemia, and high catecholamine levels frequently prompt earlier imaging, as seen in this cohort.

### Heart evaluation during donor management

LVEF assessment is essential for determining transplant eligibility.^19^ Echocardiography-guided donor management has been shown to improve cardiac function in over 50% of marginal hearts.^6^, ^10^, ^12^, ^20^, ^21^ In Switzerland, marginal hearts received earlier and more frequent echocardiography (*p* = 0.015), enabling real-time hemodynamic adjustment and multidisciplinary coordination. This strategy supports marginal heart utilization. Beyond standard imaging, advanced techniques such as strain imaging and speckle-tracking may offer deeper insights into myocardial function and help expand the donor pool.^22^, ^23^

### Donor management parameters

#### Vasopressor and pharmacologic support

BD induces neurohumoral imbalance with the development of a catecholamine storm and depletion of endogenous NE, resulting in hemodynamic instability.^24^, ^25^ In Eurotransplant data, NE ≥ 0.1 µg/kg/min was used in 58% donors in accordance with the other studies.^26^, ^27^, ^28^ Although the influence of the high-dose NE on graft success is disputable, an excessive dose of NE (>0.08 µg/kg/min) could be counterproductive for myocardial stress.^10^, ^12^

Vasopressin provides a catecholamine-sparing resuscitation without inducing bursts, with effects on V1 and V2 receptors that help stabilize hemodynamics and to treat diabetes insipidus.^29^ Supporting evidence is scant, however, and although promising, remains relatively small.^30^

Levosimendan, a long-acting calcium sensitizer, increases contractility without increased oxygen demand.^31^, ^32^ Post-transplant utilization increases extracorporeal membrane oxygenation weaning and primary graft dysfunction outcomes.^33^ However, it is not yet popular in donor optimization due to time constraints. A single case in Switzerland reported LVEF progression from 20% to 47% with levosimendan.

In Takotsubo syndrome—linked to catecholamine toxicity—levosimendan may offer benefits and could be applicable in BD-related dysfunction.^15^, ^34^

#### Volume resuscitation and hemodynamics monitoring

Adequate volume management is crucial to stabilize hemodynamics and perfuse organs in DBDs.^35^ Recommendations advocate achieving euvolemia with isotonic crystalloids.^36^ In a randomized trial (*n* = 556) investigating protocolized vs standard care, there were no differences in the number of organs transplanted (3.39 vs 3.29, *p* = 0.56) or 12-month survival, indicating that protocolized fluid resuscitation does not improve outcomes.^37^

Hemodynamic principles of monitoring are identical in critically ill patients. Both invasive and noninvasive devices—such as pulmonary artery catheters and central venous catheters—are used to assess fluid responsiveness.^36^ Nevertheless, the use of pulmonary artery catheters is quite controversial due to its possible complications, including arrhythmias and vessel damage.^38^ However, reliable monitoring of CVP, cardiac output, stroke volume, and oxygen saturation is necessary.

One study found that combining prolonged hormonal resuscitation therapy (≥15 hours) with target CVP <10 mm Hg increased heart transplants by 79%, and improved lung (95%) and kidney (13%) utilization. When both criteria were met, heart and lung procurement rates rose by 64% and 103%, respectively, without adverse effects.^39^

Dynamic indicators, such as the CVP response to fluid challenge, passive leg raising, or pulse pressure variation (PPV), are more reliable than static CVP measurements for guiding fluid therapy.^40^ Pulse contour analysis devices provide further information regarding stroke volume variation and fluid responsiveness, supporting individualized donor optimization strategies.^41^

#### Endocrine treatment

Endocrine dysfunction following BD has traditionally justified hormone replacement therapy, but not all donors exhibit true hormonal deficits, and randomized trials have not confirmed universal benefit.^36^, ^42^ Treatment should be targeted to specific deficiencies.

Diabetes insipidus occurs in 46% to 86% of donors, causing polyuria and hypernatremia, which may impair hemodynamic stability and organ viability, particularly when sodium >155 mmol/liter, increasing graft loss risk.^43^ Vasopressin is preferred over desmopressin due to its combined vascular and renal effects.^42^

Low triiodothyronine is common in brain-dead patients, often reflecting euthyroid sick syndrome. Routine thyroid hormone therapy remains controversial. While early meta-analyses found no clear benefit,^44^ some observational studies suggested improved graft outcomes.^45^ A large randomized controlled trial found no effect on heart procurement, even among donors with LVEF ≤50%,^46^ limiting indications to severe cardiac dysfunction.^47^

Central adrenal insufficiency may contribute to vasoplegia, but most donors maintain normal cortisol. Hydrocortisone has been shown to reduce vasopressor use but not improve graft outcomes; thus, routine use is not recommended but may be considered for patients with severe vasoplegia.^42^, ^48^

High-dose steroids such as methylprednisolone may reduce inflammation, but evidence remains observational, with potential adverse effects such as insulin resistance and infection.^49^

Hyperglycemia is frequent in donors and linked to poorer outcomes, though no large randomized controlled trials guide glucose management. In practice, continuing pre-BD strategies is considered reasonable.^36^

#### Resuscitation and time management

Donor hearts following cardiopulmonary resuscitation (CPR) remain viable for transplantation if adequate stabilization time is allowed.^50^ Longer post-ROSC (return of spontaneous circulation) intervals improve outcomes, particularly in cases of prolonged resuscitation. In this cohort, ROSC duration did not differ significantly between marginal and optimal hearts, but longer durations were associated with reduced echocardiographic assessment—suggesting that stress imaging and advanced monitoring could help identify transplantable grafts.

Hypoxic-ischemic brain injury is a common cause of death postcardiac arrest, often progressing to BD, thereby enabling organ donation.^51^, ^52^ In a cohort of 23,388 resuscitated patients, 13% developed BD, with higher rates following extracorporeal CPR (eCPR) compared to conventional CPR (28% vs 8.3%; *p* < 0.0001).^53^ In another study, 33% of eCPR nonsurvivors progressed to BD, and 62% became organ donors, yielding 167 transplants—predominantly kidneys and livers, with only 1 heart transplanted (1.4%).^54^ Although cardiac graft use remains rare after eCPR, these findings support its potential in expanding the donor pool.

In Switzerland, the median donor management time is 35 hours, despite protocols allowing up to 72. Extended management could improve marginal heart recovery and utilization.

#### Ex vivo perfusion and future perspectives

Ex vivo perfusion with the OCS offers advantages over cold storage by reducing ischemic time and improving postoperative outcomes.^55^ It enables prolonged preservation and enhances marginal heart utilization, as shown by Ardehali, Schroder, and Sponga.^11^, ^13^, ^56^ Future directions include ex vivo drug delivery and genetic modification to improve graft viability, potentially allowing organ importation from distant sites.^57^

### Optimized donor management and integrated protocol

This study proposes an updated protocol combining the Swiss Donation Pathway, Extended Care Bundle, and Crystal City Guidelines. Key elements include:

• *Repeated echocardiography and coronary angiography* for ongoing assessment.
• *Central hemodynamic monitoring* to guide volume and inotrope management.
• *Extended optimization periods* (up to 72 hours post-BD) as per Swiss Academy of Medical Sciences (Société Académique Suisse des Sciences Médicales) guidelines.

**Figure 3:**
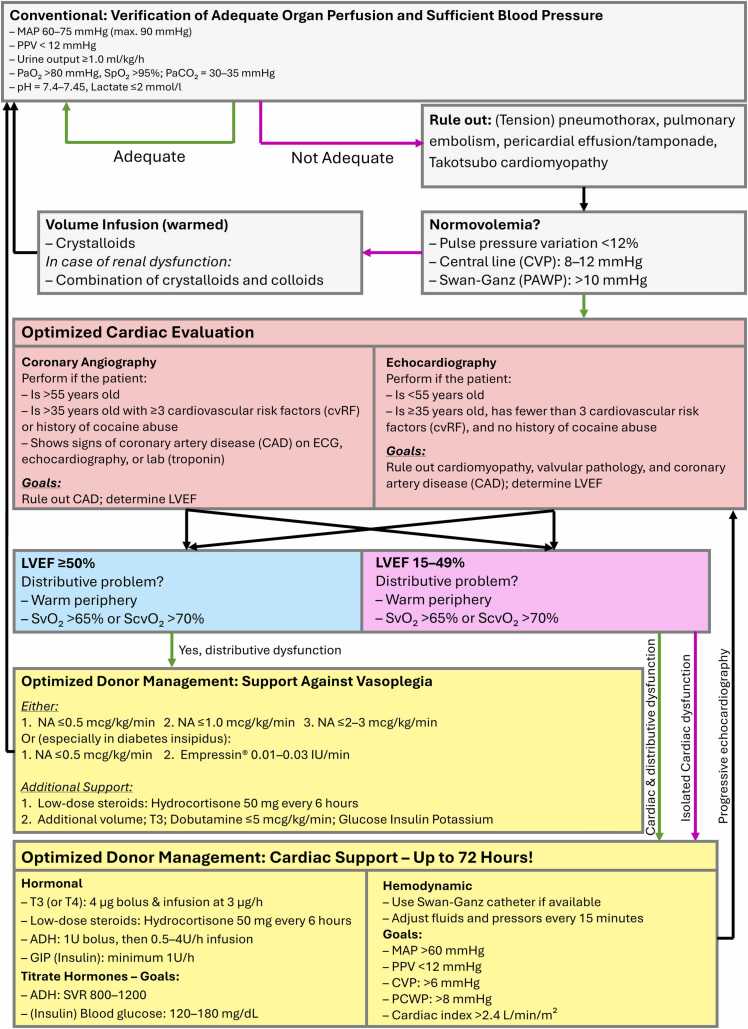
Integrated protocol for pretransplant cardiac evaluation and optimization. The flowchart integrates the Swiss Donation Pathway (2020), the Donor Optimization Extended Care Bundle (2013), the Crystal City Guidelines (2002), and findings from the present study. A red arrow indicates “no,” a green arrow indicates “yes,” and a black arrow represents the process flow. ADH, arginine vasopressin; CAD, coronary artery disease; CVP, central venous pressure; cvRF, cardiovascular risk factors; GIP, glucose-insulin-potassium; LVEF, left ventricular ejection fraction; MAP, mean arterial pressure; NA, noradrenaline; PCWP, pulmonary capillary wedge pressure; PPV, pulse pressure variation.

Figure 3 outlines a stepwise donor heart management protocol. Stabilization precedes first imaging, targeting MAP 60 to 75 mm Hg, PPV <12%, CVP 8 to 12 mm Hg, and PaO₂ >80 mm Hg. Imaging strategies are based on donor risk profile: echocardiography for low-risk, angiography for older/high-risk donors. For marginal hearts (LVEF 15%-49%), a 72-hour optimization phase with hormonal and hemodynamic therapy is recommended. Targets include MAP >60 mm Hg, CI >2.4 liter/min/m², and ScvO₂ >70%. Reassessment with repeated echocardiography and coronary angiography is advised.

## Strengths and limitations

To our knowledge, this is the first retrospective study to analyze Swiss DBD hearts (2017-2021) by categorizing them into marginal and optimal groups, and to evaluate functional recovery in marginal hearts through serial echocardiography within the framework of a nationally coordinated donor optimization system. Unlike previous studies focusing solely on graft outcomes or donor selection criteria, we report dynamic improvements in LVEF over time and identify actionable management variables, such as vasopressor use, volume management, and resuscitation, associated with transplant eligibility. This structured approach supports a shift toward evidence-based expansion of marginal donor utilization. However, limitations include the retrospective design, lack of standardized protocols, and group differences that reduced statistical power. Regarding the remaining marginal hearts, for those without repeated echocardiography (*n* = 47), LVEF was assessed based on a single echocardiogram. This limitation may introduce a selection bias, as follow-up imaging was more likely pursued in donors considered to have transplant potential. Ischemia management was not evaluated, and data on retrieval centers were not analyzed, limiting the scope of conclusions. Moreover, this study did not assess postoperative recipient outcomes or graft function beyond the point of organ allocation. As a result, our primary end-point was the utilization of donor hearts for transplantation, not the clinical success of the transplant. Future analyses incorporating post-transplant outcomes would be valuable to further validate the findings of this study and assess the long-term viability of functionally marginal hearts. Finally, another limitation lies in the lack of ischemic time data and detailed perfusion-hemodynamic monitoring parameters across all donors. These variables were not consistently reported across centers and years, limiting their inclusion in our statistical models. Additionally, no donors in this cohort were managed using ex vivo organ perfusion platforms such as the OCS.

## Conclusion

This study underscores the importance of optimized donor management in increasing the use of marginal hearts. Improved LVEF, particularly in structurally normal donors, was strongly linked to higher transplantation rates. Pharmacologic support with NE and vasopressin, alongside endocrine and volume resuscitation strategies, contributes to hemodynamic stability and graft viability.

Emerging tools such as ex vivo perfusion (OCS) and advanced imaging (echocardiography, angiography) enhance donor evaluation and preservation. Extending optimization duration and standardizing protocols may further improve outcomes.

Future efforts should focus on refining therapies and expanding marginal donor criteria. Through these strategies, heart transplant programs can increase donor utilization and improve patient survival.

## Ethics Statement

In the opinion of the Ethics Committee of the Canton of Bern (Req-2025-00514), the planned project is not subject to the Swiss Human Research Act and thus does not need a prospectively obtained approval from the responsible ethics committee.

## Funding

None declared.

## CRediT authorship contribution statement

S.A. and F.I. contributed to the conception and design of the study. S.A., R.G., and F.I. collected and curated the data. Data analysis and interpretation were performed by R.G., S.A., and F.I. R.G., S.A., K.B., B.A., and F.I. drafted the manuscript. R.H. and F.B. provided critical revisions and intellectual input. All authors reviewed and approved the final version of the manuscript.

## Declaration of Competing Interest

The authors declare that they have no known competing financial interests or personal relationships that could have appeared to influence the work reported in this paper.
